# Evaluation of a novel fixed-space maintainer made of light-cured acrylic resin: an in vitro study

**DOI:** 10.1038/s41405-020-00046-1

**Published:** 2020-09-23

**Authors:** Yasser R. Souror, Tayseer Maaly, Mohammed Sameer Khawandanah

**Affiliations:** 1 Department of Pediatric Dentistry, Faculty of Dentistry, Al-Azhar University, Assuit, Egypt; 2 Pediatric Dentistry Department, Batterjee Medical College, Jeddah, Saudi Arabia; 3 Department of Dental Material, Faculty of Dentistry, Zagazig University, Zagazig, Egypt; 4 Dental Intern, Department of Dentistry, Batterjee Medical College, Jeddah, Saudi Arabia

## Abstract

To evaluate a fixed-space maintainer made of light-cure acrylic resin (LCAR) for its flexural and shear bond strength using different bonding systems to the enamel. 45 extracted primary teeth were selected. They were randomly divided into three equal groups (*n* = 15) along with the type of adhesive system (Tetric Flow, Transbond XT, and Fuji Ortho LC) used for bonding (LCAR) to the tooth surface. Surfaces were treated; LCAR was attached to the treated surfaces using a split Teflon mold. For flexural strength testing, ten bars of LCAR were made using another Teflon-split mold. Shear bond strength and mean flexural strength values were evaluated by a universal testing machine. The highest values of bond strength were recorded for Transbond XT, followed by Tetric Flow, while the lowest values were for Fuji Ortho LC. Various groups had a significant difference as investigated by ANOVA. ARI scores showed no significant difference in debond sites. Mean value and standard deviation of flexural strength for LCAR were 82.83  ± 5.2. LCAR has superior mechanical properties and could be an alternative to currently-in-use space maintainer though in vivo and in vitro trials are needed to progress the ultimate design of LCAR.

## Introduction

Untimely loss of primary molars may produce teeth movement, leading to loss of space and arch deficiency.^1^ Following this, space loss could produce or exaggerate existing malocclusions, such as crowding, ectopic eruption, extreme both overjet and overbite, and opposed molar contacts.^2^ The amount of space loss in the mandible is greater than that lost in the maxilla next to the lost primary tooth.^3,4^ However, after premature loss of the primary second molar in the early mixed dentition stage, the space loss has been reported to be very large in either maxilla or mandible.^5^

To limit the decrease in dental arch diameter, by preserving a relative location of the current teeth, a fixed or removable space maintainer is employed next to the lost primary tooth.^6,7^ A removable space maintainer is simple to construct and re-establishes functions and aesthetics. However, removable space maintainers are more frequently lost than fixed-space maintainers.^8^ Fixed-space maintainers, such as Band and Loop, have a good success rate. However, cement fragmentation, solder breakage caries formation along the borders of the band, and a long assembly time are some of the disadvantages associated with them. It is considered a nonaesthetic appliance due to its metallic appearance.^9^

Fiber-reinforced composite is an alternative aesthetic space maintainer, and although the material has an acceptable appearance, the material is flexible with a lack of rigidity, ending in a larger amount of dislocation for a certain amount of force compared to Band and Loop.^10^

Acrylic-based light-cured materials are common in dental applications. Common applications of LCAR include custom trays, temporary crowns, and removable orthodontic appliances.^11,12^

The basic in vitro tests employed to test clinical acceptance are flexural and shear bond strength. The ability of the material to bend before fracture is defined as flexural strength. It is attained as soon as the definitive elasticity of one material is accomplished compared to its proportional limit.^13^ Dental materials must exhibit enough flexural strength, to bear repeated flexing, bending, and twisting in clinical situations. Materials with low flexural strength are more liable to permanent deformation when subjected to the action of chewing stress.^14^

In clinical situations, adhesive materials must have high bond strength to resist different types of stresses. Contraction of the resin is the reason for these stresses, which when loaded in the bond region of tooth and restoration for extended times, can lead to failure. Failure of the adhesion between the tooth and restoration leads to many problems, such as failure of the restoration, sensitivity, and tooth-recurrent caries.^15^

Our study aimed to evaluate a fixed-space maintainer made of light-cured acrylic resin regarding its flexural and shear bond strength using various bonding systems.

## Materials and methods

### I-**Shear bond strength testing**

1-**Preparation and grouping of specimens**

In this study, we used sample size used in the previous study conducted on the same test.

Forty-five extracted lower second primary molars were selected in this study.

Inclusion criteria:

Intact buccal and lingual surfaces.

No decay, cracks, or defects.

The samples were cleaned by tap water and a soft brush for 1 min. Then, all samples were examined under a stereomicroscope (JSM-5400, JEOL, Japan) for any surface cracks or fracture.^16^ The sound teeth were prepared with pumice slurry (Nada^™^ Pumice Paste—Fluoride Free Saint Paul, MN 55120), and rubber cup (Screw style, flat Prophy Cup Silicon Model No. PC-310 China) and then they were kept in 0.5% chloramine-T solution (Fisher chemical, Fair lawn, NJ, USA) at 4 °C for 24 h.^17^

The materials used in the study are presented in Table 1.

**Table 1:** Used materials in the study.

Material	Manufacturer	Chemical composition
Triad (VLC)	Dentsply, York, PA	Urethane dimethacrylate, silica, and PMMA beads
Transbond XT	3M Unitek, Monrovia, CA,USA	Bis-phenol A glycidylmethacrylate, quartz, and submicron silica
Tetric flow^TM^	Ivoclar-Vivadent, Schaan, Liechtenstein	Bis-phenol A glycidylmethacrylate, urethane dimethacrylate, barium glass, and ytterbium trifluoride
Fuji Ortho LC	GC America Inc., Tokyo, Japan	Aluminosilicate glass, polyacrylic acid, hydroxyethyl methacrylate, and triethylene glycol methacrylate

The root portion of the teeth was removed using a diamond saw (BRASSELER USA DIAMOND Brasseler U.S.A. Dental, LLC).

**Fig. 1:**
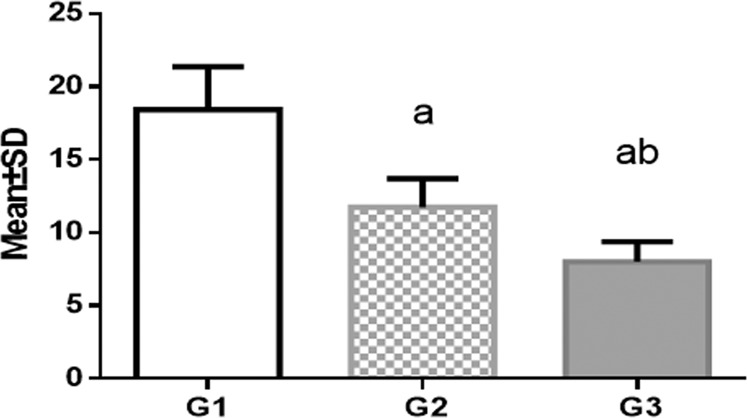
Shear bond strength values. Post hoc grouping. Different letters indicate significant difference.

**Fig. 2:**
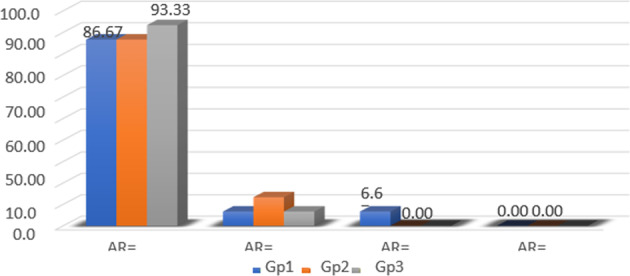
The distribution of ARI in relation to the samples in each group. ARI = zero in most of the samples. Gp1, Gp2 and GP3; Group1, Group2 and Group3.

After proportioning of the powder and liquid of cold-cured acrylic resin (335201 GC Corporation Tokyo 113-0033, Japan) was done according to the manufacturer’s instruction, the mix was packed in a plastic tube (2-cm diameter, 2-cm length); then, the crown portions of the teeth were horizontally mounted on the acrylic resin exposing the facial surface outward.^16^ By using a diamond fissure bur (Swiss Goldie Flat Fissure (SG 835, Dental Brands Australia)), buccal enamel surfaces were superficially prepared under air–water coolant spray to reach a smooth surface (Figs. 1 and 2). The teeth were randomly separated into three groups (each containing 15 specimens), depending on the type of bonding the adhesive system for 40 s.

Group 1: phosphoric acid etchant (Super-etch) (Phosphoric acid 37%, SDI, Australia) was spread over the enamel surface for 15 s and washed for 15 s. Excess water was eliminated using a spongy pellet, to get a moist enamel surface. Two layers of the single-bond adhesive (Adper Single Bond-2^®^ 3M) were added to the etched enamel surface using a brush and cured for 20 s by using a light-curing device with 450 mW/cm^2^ intensity for 20 s (Litex 680 A, Dentamerica, California, USA). A thin coat of resin cement Transbond XT (3M Unitek, Monrovia, CA, USA) was spread to the surface of the tooth and cured for 40 s with the visible-light-cure unit.

Group 2: phosphoric acid etchant (Super-etch) was spread over the enamel surface for 15 s and then washed for 15 s. Excess water was eliminated using a porous pellet to get a moist enamel surface. Two layers of the single-bond adhesive (Adper Single Bond-2^®^ 3M) were applied to an etched surface using a disposable brush and light-cured for 20 s. A thin layer of flowable composite Tetric flow^TM^ (Ivoclar-Vivadent, Schaan, Liechtenstein) was added on the tooth surface and then cured for 40 s.

Group 3: phosphoric acid etchant (Super-etch) was added for 15 s on the enamel surface and rinsed for 15 s. Excess water was eliminated using a soft pellet, leaving the enamel surface wet. Resin-modified glass ionomer capsule Fuji Ortho LC (GC America Inc., Tokyo, Japan) was triturated for 10 s, the capsule was applied in a gun, and then the cement was placed on etched enamel and then light-cured for 40 s.

#### Application of light-cured acrylic resin

Triad VLC was introduced into the central hole of the Teflon mold with an internal diameter of 3 mm and height of 4 mm, in incremental layers, filled up to the edge of the mold, so after curing of the resin, all the bars were of 3-mm diameter and 4-mm height with no need for trimming.^16^ Each increment was light-cured for 2 min. The Teflon mold was detached, and LCAR was polymerized again for an extra 2 min to ensure complete polymerization. Specimens were saved in 37 °C water for 24 h and then thermocycled with a range of 5–55 °C.

#### Testing

Shear bond strength test was evaluated by a Testing Machine (LLOYD instruments, LR 5K, England). The blade directed corresponding to the bonding surface at resin–enamel interface, the force was applied to the specimens at 0.5 mm/min, a cross-head speed.^16^ Values were estimated in Newton, the highest load of failure divided by the bond surface area (mm^2^) and converted into megapascal (MPa). The results were tabulated and statistically analyzed. ANOVA was used to relate the different bond strength values for different groups. Adhesive remnant index (ARI)^18^ was used to weigh the adhesive quantity remaining on enamel. There are four scales, ranging from 0 to 3. No material left on the tooth is 0; when adhesive covered lower than half the bonded area, the scale is 1; further than hemi-bonded area enclosed by an adhesive, the scale is 2; when adhesive covered the total bonded area, the scale is 3.

### Flexural test

#### Specimen preparation

Ten specimens were measured (16 × 5 × 4 mm),^19^ prepared by condensing LCAR in a white polytetrafluoroethylene split mold against a microscope glass slab. The uncured LCAR was pressed between two glass slabs to the thickness of the mold, which inserted in an adjustable frame.^19^ Tahe test specimens were set by a light-curing unit (Litex 680 A, Dentamerica, California, USA) with the 450 mW/cm^2^ intensity for 2 min from both the sides. Then, the mold was removed, and LCAR was cured for an extra 2 min again to ensure complete polymerization. Specimens were set aside in water at 37 °C for 48 h earlier to evaluation.

#### Testing

A three-point evaluated design was used for detecting mean flexural strength values; the specimen was loaded at 1 mm/min cross-head speed of the force directed centrally over a distance of a two-point support. Universal Testing Machine (LLOYED instruments, LR 5K, England) was used. The specimens were deflected, and force of break was recorded. The stress was estimated by the equation: *S* = 3·*P*·*L*/2·*b*·*d*2, expressed in MPa, where *P*, expressed in *N*, is the load at a given point on the curve of the load-deflection curve; *L*, expressed in mm, length of the support; *b*, expressed in mm, is the beam width; *d*, expressed in mm, is the tested beam depth.^19^

### Statistical analysis

The data were analyzed by software computer program version 23 (SPSS, Inc., Chicago, IL, USA). Values were stated in mean and standard deviation. One-way analysis of variance (ANOVA) and post hoc Tukey test were used for comparing more than two dissimilar groups. Kolmogorov–Smirnov was used to asses normality of distributions. There are statistically significant deference outcomes when *p* value is <0.05. Chi-square was utilized to confirm substantial differences for ARI scales among various materials, statistically significant, when *p* value is <0.05.

## Results

### Flexural strength result

Minimum, median, and maximum values were 75.78, 82.83, and 90.07, respectively. The mean value and standard deviation of flexural strength for Triad (VLC) was 82.83 ± 5.2.

### Shear bond strength results

The mean and standard deviation values of bond strength for all groups exist in Table 2 and Fig. 1. ANOVA indicated significant difference among the various groups as shown by (*p* < 0.05). Transbond XT cement recorded the highest bond strength values (18.43 ± 2.93). Fuji ortho LC recorded the lowest value (8.02 ± 1.37); Tetric Flow exhibited significantly increased shear bond strength values (11.47 ± 2.74) over Fuji ortho LC and lower than Transbond XT (*p* < 0.05). The ARI results for the various groups are shown in Table 3 and Fig. 2. The results of chi-square test specified no significant differences in deboned area (ARI score) between various groups (*p* > 0.05).

**Table 2:** Shear bond strength results.

Material	Mean ± SD	*p* value
G1: Transbond XT	18.43 ± 2.93	*p* < 0.05
G2: Tetric Flow	11.47 ± 2.74^a^	
G3: Fuji Ortho LC	8.02 ± 1.37^ab^	

^a^Significant difference between G1 and G2.

^ab^Significant difference between G3 and both G1 and G2.

**Table 3:** ARI score frequency of distribution.

Scale	Group 1	Group 2	Group 3	*X*^2^	*p* value
AR = 0	13 (86.7%)	13 (86.7%)	14 (93.3%)	2.55	0.63
AR = 1	1 (6.7%)	2 (13.3%)	1 (6.7%)		
AR = 2	1 (6.7%)	0 (0%)	0 (0%)		
AR = 3	0 (0%)	0 (0%)	0 (0%)		

## Discussion

Fixed-space maintainers are important for the preservation of the integrity of the primary dental arch; Band and loop appliances are strong and well tolerated; however, they have some disadvantages: they do not restore the typical function, unaccepted metallic appearance, intermittent removal of the appliance for fluoride application, checking, and cleaning. In addition, they require clinical and laboratory steps for their manufacture.^2^

Light-cure acrylic resins are gaining popularity for dental applications. It is a well-established option for prosthodontic and orthodontic appliances.^20^ Triad VLC is one type of light-cured acrylic resin; it is a colored material and composed of urethane dimethacrylate in addition to reinforcement with glass fibers. Since the longevity of using Triad VLC as a space maintainer is a controversial issue, this study aimed to measure its flexural and shear bond strength using different adhesive systems.

The aim of the shear bond strength test is to assess bond strength value and it is defined as maximum force that a bonded area can bear before failure.^21^ LCAR bonded to the tooth by Transbond XT bonding system, exhibited increased shear bond strength over that bonded by Tetric Flow or Fuji Ortho LC, and significant difference among the test groups was recorded (*p* < 0.05). This finding agrees with a previous study^22^ which showed that fiber-reinforced composite produced significantly higher shear bond strength when bonded using Transbond XT than Tetric flow and Fuji Ortho LC. Fiber-reinforced composites are fitting matrices that embed axial particulates, and they are used for many dental applications, such as prosthodontics, conservative dentistry, and orthodontics.^23^

Transbond XT cement was introduced for the adhesion of orthodontic accessories to enamel. It is an ideal standard, commonly used in shear bond strength research, which evaluated the orthodontic bonding success of different products.^24^ Short manipulation time, no necessity for mixing, and enough adhesion to tooth surfaces are the main advantages offered by Transpond XT composite.^25,26^ So it is being mainly applied in clinical orthodontics and used as standard in experimental studies. In the present study, this composite produced a mean shear bond strength value of 18.43 ± 2.93, which confirms its good adhesiveness to dental enamel.^27^

Flowable composites are formulated by reducing filler content and increasing resin content, resulting in low-viscosity resins, keeping the same particle size of a traditional hybrid composite.^28^ Due to its low viscosity, flowable composites flow and adjust to the restoration. At the same time, the sliding movement of material owing to gravity is a serious shortcoming of flowable composite, resulting in poor adaptation. Increased viscosity of Transbond XT paste allowed restoration to be sited over the tooth surface in the favorite situation.^29^ This may explain the higher bond strength of LCAR with Transbond XT compared to flowable composites.

The bond of glass ionomer cements with enamel is an ionic chemical in nature. It is created when the calcium ions of the tooth, which are positively charged, react with negatively charged carboxylic groups in GICs. To achieve high bond strength, polyacrylic acid is applied onto the enamel surface. The effect of this weak acid is to freshen the tooth surface only without any demineralizing effect.^30^ This effect produced by phosphoric acid etching is not suitable for GICs, as it results in subsequent reduction in bond strength. For this reason, GICs are not completely accepted as a gold standard adhesive for orthodontic appliances.^31^ However, a previous study^32^ stated that the bond strength ranging from 18.9 MPa was for verified bonding of RMGIs to enamel; thus, it may be appropriate for attachment of the orthodontic bracket and space maintainer to the tooth. Yassaei et al. evaluated the similar materials of our study for shear bond strength testing, and they found that composite resin recorded higher bond strength in comparison with RMGI, and the result was significantly different, which is agreeing with our results.^33^

It was found that the range of 6 and 8 MPa is the lowest bond strength required for bonding different orthodontic appliances. This value is suitable for most clinical orthodontic requirements because they may be able to resist orthodontic forces and ones of mastication.^34^ This study stated that the bond strengths of test adhesives were above that value. In fact, adhesion forces should not be too strong in order to avoid enamel loss after debonding (40–50 MPa).^35^

Therefore, an ideal orthodontic biomaterial should have bonding forces included in the interval of 5–50 MPa.

Following debonding of LCAR from tooth surface, ARI scores were evaluated to confirm the sum of the adhesive remaining on enamel. In previous studies,^36^ involving adhesives of bonding the orthodontic accessories, the results of bond strength data did not typically match the results of ARI. In our study, statistically significant differences among the values of bond strength did not match ARI rank, as shown in Tables 2 and 3, respectively. Failed locations showed no significant differences in the ARI score between the different groups. ARI score 0 was the frequency for all groups, so there is no adhesive persisting on the tooth in the bond location.

It is also important to evaluate materials’ flexural strength. This is a blend of compressive, tensile, and shear strengths; the different strengths reproduce the rigidity and resistance of a material to break.^37^ Our study evaluated flexural strength for LCAR, by the three-point bending experiment. The test is widely used in dental research, because it has many advantages, such as simple load application, simple specimens’ fabrication, and similar stress distribution to that in fixed bridges.^38^

Specimens of the present studies were fabricated using a split Teflon mold with dimensions 16 × 4 × 5 mm; these dimensions are clinically realistic dimensions for the flexural as recommended by previous study.^39^ Lesser dimensions of specimens resulted in values close to that attained with the identical specimen (ISO 4049), but with saving time and less amount of material.^39^ In this study, specimen dimensions were with a greater cross section, similar to the clinical situation and shorter than standard.^19^ For screening of resin-based materials, flexural strength is an important mechanical property, chosen by the International Organization for Standardization. It must be more than 50 MPa for crown and bridge made of polymer materials.^40,41^ Our study evaluated the flexural strength of fixed-space maintainer made of LCAR; the mean flexural strength was 80.22 ± 9.99 MPa. This finding was consistent with a former study,^41^ where the mean value of flexural strength of LCAR was 82.83 ± 5.2 MPa.

## Limitations

1. The current study does not verify bond strength of LCAR to the permanent teeth.
2. The biological considerations in terms of plaque accumulations and bacterial colonization were not measured.
3. It was an in vitro study to test some properties of a material that could be used as a space maintainer, and further biological and lasting clinical evaluation is needed to develop the best design of LCAR space maintainer.

## Conclusions

Within the restrictions of our research, the LCAR has an accepted flexural and shear bond strength to enamel, which may qualify to be used as a fixed chair-side space maintainer in primary dentition.
